# Die Entwicklung der Mittelschicht in Österreich und Deutschland

**DOI:** 10.1007/s10273-022-3293-2

**Published:** 2022-10-20

**Authors:** Dénes Kucsera, Hanno Lorenz, Wolfgang Nagl

**Affiliations:** 1 Agenda Austria, Türkenstraße 25/9-10, 1090 Wien, Österreich; 2 Fakultät für Angewandte Wirtschaftswissenschaft, THD - Technische Hochschule Deggendorf, Dieter-Görlitz-Platz 1, 94469 Deggendorf, Deutschland

**Keywords:** D31, D63, H24, O15

## Abstract

Wir untersuchen die Mittelschicht in Österreich und Deutschland, wobei ein besonderer Fokus auf den Veränderungen der vergangenen 20 Jahre liegt. Es wird gezeigt, dass sich die Mittelschicht in Deutschland und Österreich in ihrer Zusammensetzung hinsichtlich Bildung, Familienkonstellationen und Alter verändert hat, aber immer noch in beiden Ländern, in Österreich etwas mehr als in Deutschland, den Großteil der Bevölkerung umfasst. Anschließend analysieren wir die Bedeutung der Mittelschicht für den Sozialstaat beider Länder.

## Abstract (en)

We examine the changes and evolution of the middle class in Austria and Germany over the past 20 years. During that period, the middle class in Germany and Austria has changed in terms of education, family constellations and age. In both countries, however, a large part of the population still belongs to the middle class. In Austria, the percentage of people in the middle class appears to be stable and is slightly higher than in Germany. During the COVID-19 crisis, discretionary policy measures have been effective in limiting welfare losses for the middle class. Finally, we analyse the importance of the middle class for the welfare state of both countries.

Menschen verorten sich oft in der Mittelschicht der Gesellschaft, obwohl sie, gemessen an ihrem Einkommen oder Vermögen, höheren oder niedrigeren Einkommensschichten zuzuordnen wären (Fessler et al., 2019; Niehues und Stockhausen, 2019). Eine breite Mittelschicht ist aber nicht nur individuell erstrebenswert, sondern auch von gesamtgesellschaftlicher Relevanz. So zeigt sich, dass in Ländern mit einer breiten Mittelschicht die soziale Mobilität höher ausgeprägt ist (OECD, 2018). Menschen, die zur Mittelschicht gehören, investieren mehr in ihre Bildung, stehen entsprechend als gut ausgebildete Fachkräfte zur Verfügung und tragen auch positiv zu Innovationen und Unternehmertum bei (Brücker et al., 2018; Chun et al., 2011; Doepke und Zilibotti, 2005). Zudem wirkt eine breite Mittelschicht auch in Krisenzeiten stabilisierend: So waren in der vergangenen Wirtschafts- und Finanzkrise die Einkommensverluste der Mittelschicht geringer als jene der niedrigeren und höheren Einkommen (OECD, 2019).[1] Insgesamt wachsen die Einkommen in der Mittelschicht aber langsamer als die der oberen 10 % der Einkommensverteilung, aber auch deutlich stärker als die der unteren 10 % (OECD, 2019).

## Abgrenzung und Definition der Mittelschicht

Die Mittelschicht kann auf Basis des Vermögens oder anhand des Einkommens definiert werden (Piketty, 2018; OECD, 2019). Die entscheidende Größe zur Finanzierung des alltäglichen Lebens ist das verfügbare Einkommen. Entsprechend wird dieses zumeist herangezogen, um die Mittelschicht einzugrenzen (Grabka et al., 2016; Ravallion, 2010; Atkinson und Brandolini, 2013; OECD, 2015; Bundesministerium für Arbeit, Soziales und Konsumentenschutz, 2017).

Aufgrund der unterschiedlichen Haushaltsgrößen und einer besseren Vergleichbarkeit bedarf es einer Äquivalisierung des verfügbaren Einkommens. Es werden also für eine Lebensgemeinschaft die gesamten Bruttoeinkünfte aus Arbeit und Kapital abzüglich der Steuern und Sozialabgaben und zuzüglich aller öffentlicher Transfers addiert. Dieses verfügbare Haushaltseinkommen wird auf alle Mitglieder eines Haushalts gemäß des Anpassungsfaktors der OECD, der das Haushaltseinkommen mit der Quadratwurzel der Haushaltsmitglieder gewichtet, verteilt.[2]

1 Eine breite Datenbasis der Auswirkungen der Coronakrise auf die Mittelschicht liegt noch nicht vor.

2 Neben dem Anpassungsfaktor der OECD wird häufig auch noch die sogenannte EU-Skala für Äquivalisierung der Einkommen auf Haushaltsebene verwendet. Der ersten Person im Haushalt wird dabei das Gewicht 1,0 zugewiesen. Jede weitere Person mit mindestens 14 Jahren erhält das Gewicht 0,5, jeder Person jünger als 14 Jahre erhält das Gewicht 0,3.

**Dr. Dénes Kucsera** und **Hanno Lorenz** sind Ökonomen bei der Agenda Austria in Wien.

**Prof. Dr. Wolfgang Nagl** ist Professor für Volkswirtschaftslehre mit Schwerpunkt Wirtschaftspolitik an der Technischen Hochschule Deggendorf.

Tabelle 1
**Ober- und Untergrenzen des Einkommens für die Zugehörigkeit zur Mittelschicht**

| | Österreich | | | | |
|---|---|---|---|---|---|
| | Einpersonenhaushalt | Paarhaushalt | Familie mit einem Kind | Familie mit zwei Kindern | Familie mit drei Kindern |
| Untergrenze (in Euro) | 22.193 | 31.386 | 38.440 | 44.386 | 49.625 |
| Obergrenze (in Euro) | 59.067 | 83.533 | 102.306 | 118.133 | 132.077 |
| | Deutschland | | | | |
| | Einpersonenhaushalt | Paarhaushalt | Familie mit einem Kind | Familie mit zwei Kindern | Familie mit drei Kindern |
| Untergrenze (in Euro) | 18.536 | 26.214 | 32.105 | 37.072 | 41.448 |
| Obergrenze (in Euro) | 49.419 | 69.889 | 85.596 | 98.838 | 110.504 |

Die Untergrenze (Obergrenze) definiert sich bei 75 % (200 %) des mittleren verfügbaren äquivalisierten Haushaltseinkommens. Für Österreich sind alle monetären Werte in Euro des Jahres 2019 ausgedrückt. Für Deutschland in Euro des Jahres 2018.

Quelle: LIS Cross-National Data Centre (2022).

Nach der Berechnung des äquivalisierten Einkommens muss die Mittelschicht definiert werden. In einem ersten Schritt wird das Medianeinkommen aus den äquivalisierten verfügbaren Einkommen ermittelt. Auf Basis der von uns herangezogenen Mittelschichtdefinition der OECD (2019) umfasst die Mittelschicht alle Personen, deren verfügbares Einkommen zwischen 75 % und 200 % des mittleren Einkommens liegen.[3]

## Veränderungen in der Mittelschicht in Österreich und Deutschland

Um Veränderungen und Dynamiken in der Mittelschicht detaillierter zu identifizieren, werden die Daten der Luxembourg Income Study Database (LIS) herangezogen. Die LIS beinhaltet harmonisierte Daten unter anderem bezüglich Arbeits- und Kapitaleinkommen, Renten und Pensionen, öffentliche und private Transferleistungen und Steuern und Sozialversicherungsabgaben von über 50 Ländern und einen Zeitraum von über 50 Jahren.

Auf Basis der Mittelschichtdefinition der OECD ergeben sich für die verschiedenen Haushaltstypen in Österreich und Deutschland die folgenden Einkommensunter- und Einkommensobergrenzen (vgl. Tabelle 1). So gehört in Österreich ein Singlehaushalt mit einem verfügbaren Einkommen zwischen 22.193 Euro und 59.067 Euro der Mittelschicht an. Bei einer klassischen Familie mit zwei Kindern steigen diese Grenzen auf 44.386 Euro und 118.133 Euro an. In Deutschland ist die Mittelschicht stärker konzentriert und weniger breit. Als Single erreicht man diese bereits mit 18.536 Euro und verlässt die Mitte in Richtung Oberschicht bei 49.419 Euro. Bei der klassischen Familie mit zwei Kindern liegen die Grenzen des verfügbaren Einkommens bei 37.072 Euro und 98.838 Euro.

Die historische Entwicklung der Mittelschicht in Österreich und Deutschland zeigt, dass deren Einkommen mit der Zeit ansteigen. In Österreich und Deutschland hat sich im Zeitablauf die Ober- und Untergrenze – selbst inflationsbereinigt – nach oben verschoben. So lag in Österreich im Jahr 2000 die Untergrenze der Mittelschicht bei 18.805 Euro und die Obergrenze bei 49.847 Euro.[4] 2019 lag die Untergrenze bei 22.193 Euro und die Obergrenze bei 59.067 Euro. In Deutschland haben sich die Grenzen von 16.803 Euro und 44.800 Euro im Jahr 2000 auf 18.536 Euro und 49.419 Euro im Jahr 2018 erhöht.[5] Es lässt sich entsprechend konstatieren, dass die realen Einkommen der Mittelschicht in beiden Ländern über die Zeit gestiegen sind.

Ein Vergleich über die Zeit zeigt, dass die realen äquivalisierten Haushaltseinkommen in Österreich (vgl. Abbildung 1) und Deutschland (vgl. Abbildung 2) in den vergangenen 20 Jahren gestiegen sind und sich die entsprechenden Einkommensverteilungen nach rechts verschoben haben. So ist das inflationsbereinigte äquivalisierte Medianeinkommen in Österreich von rund 25.073 Euro im Jahr 2000 auf 29.591 Euro im Jahr 2019 gestiegen. In Deutschland lag das inflationsbereinigte äquivalisierte Medianeinkommen im Jahr 2000 bei 22.404 Euro und im Jahr 2018 bei 24.715 Euro.[6]

Da die Mittelschicht als relatives Maß definiert ist, sagt eine Rechtsverschiebung der Einkommensverteilung und ein Anstieg des Medianeinkommens nicht direkt etwas

3 Es existieren noch zahlreiche andere Abgrenzungen der Mittelschicht. Diese unterscheiden sich hauptsächlich hinsichtlich der Einkommensgrenzen rund um ein äquivalentgewichtetes mittleres Einkommen (Atkinson und Brandolini, 2013; Goebel et al., 2010; Grabka et al., 2016; OECD, 2015; Ravallion, 2010; Bundesministerium für Arbeit, Soziales und Konsumentenschutz, 2017).

4 Gemessen als verfügbares äquivalisiertes Haushaltseinkommen in Euro des Jahres 2019.

5 Gemessen als verfügbares äquivalisiertes Haushaltseinkommen in Euro des Jahres 2018.

6 Es wurde das jeweils aktuell verfügbare Jahr verwendet.

Abbildung 1
**Verteilung des verfügbaren äquivalisierten Haushaltseinkommens in Österreich**

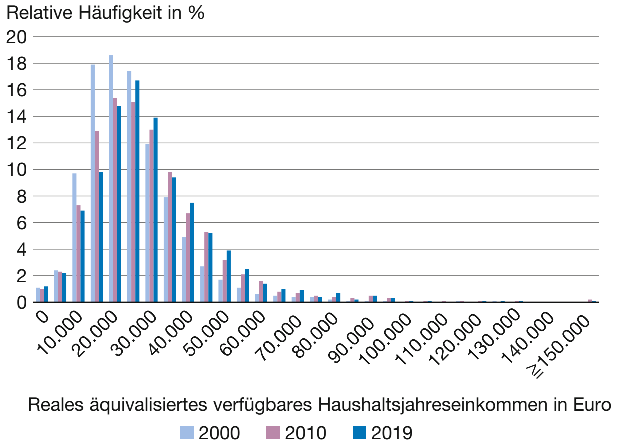

Die verfügbaren äquivalisierten Haushaltseinkommen sind zum Basisjahr 2019 inflationsbereinigt. Die Berechnungen erfolgen in 5.000-Schritten. Die höchste Gruppe umfasst alle Haushaltseinkommen von 150.000 und darüber.

Quellen: LIS Cross-National Data Centre (2022); Statistik Austria (2022); eigene Berechnungen.

Abbildung 2
**Verteilung des verfügbaren äquivalisierten Haushaltseinkommens in Deutschland**

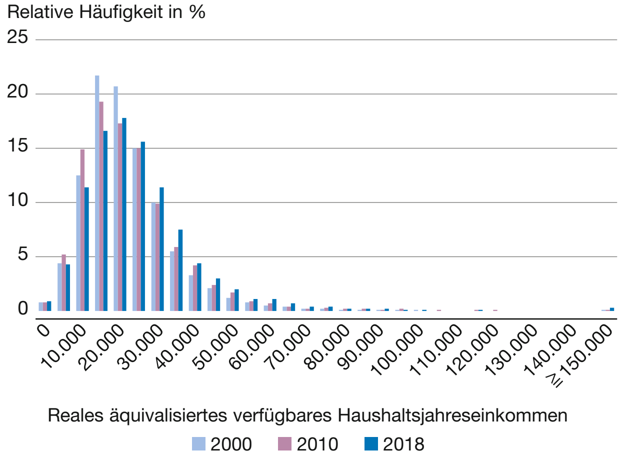

Die verfügbaren äquivalisierten Haushaltseinkommen sind zum Basisjahr 2018 inflationsbereinigt. Die Berechnungen erfolgen in 5.000-Schritten. Die höchste Gruppe umfasst alle Haushaltseinkommen von 150.000 und darüber.

Quellen: LIS Cross-National Data Centre (2022); Statistisches Bundesamt (2022); eigene Berechnungen.

über die Größe der Mittelschicht aus. Betrachtet man die Entwicklung der Mittelschicht explizit über die Zeit, zeigt sich, dass in der Alpenrepublik der Anteil der Menschen, die zur Mittelschicht gehören, in den vergangenen 20 Jahren annähernd konstant zwischen 65,8 % und 68,9 % lag. In Deutschland lag der Anteil der Mittelschicht im Jahr 2000 noch bei knapp unter 70 %. Seit 2005 liegt dieser mit rund 64 % etwas darunter. Gleichzeitig stieg der Anteil der Personen unter 75 % des Medianeinkommens leicht an. In Österreich blieb der Anteil der Personen mit weniger als 75 % des Medianeinkommens weitgehend konstant. Der Anteil der sehr einkommensstarken Personen mit über 200 % des Medianeinkommens ist in beiden Ländern relativ stabil (vgl. Tabelle 2).

Abseits der Größe der Mittelschicht hat sich die soziodemografische Zusammensetzung über die Zeit wesentlich verändert. Mit dem demografischen Wandel finden sich in der mittleren Einkommensschicht in beiden Ländern immer mehr ältere Personen. Der Anstieg ist in Deutschland ausgeprägter als in Österreich, was an der in Deutschland stärker ausgeprägten Alterung der Gesellschaft liegen mag (vgl. Tabelle 3). Spiegelbildlich geht in beiden Ländern der Anteil der jüngeren Kohorten an der Mittelschicht zurück.

Die dominante Haushaltsform der Mittelschicht ist nach wie vor das Paar mit Kindern. Der Anteil ist allerdings rückläufig. So lag dieser im Jahr 2000 in Österreich noch bei 52,9 % und in Deutschland bei 50,8 % (vgl. Tabelle 3). Am aktuellen Rand liegt der Anteil nur noch bei 47,6 % in Österreich und 44,0 % in Deutschland. Gleichzeitig ist der Anteil der Einpersonenhaushalte und der Paare ohne Kinder angestiegen.

In Österreich ist in den vergangenen Jahrzehnten das durchschnittliche formale Bildungsniveau in der Mittelschicht gestiegen.[7] Der Anteil an Personen mit mittlerem Qualifikationsniveau in der Mittelschicht ist gleichgeblieben. Personen mit niedrigem Bildungsniveau waren seltener in der Mittelschicht zu finden, Personen mit hohem Bildungsabschluss dafür häufiger. So hat sich in Österreich der Anteil an Personen mit hoher Bildung von 8,7 % auf 17,8 % mehr als verdoppelt. In Deutschland wiederum ist der Anteil an Personen mit hoher Bildung in der Mittelschicht von einem deutlich höheren Niveau aus noch weiter gestiegen. Fast jede dritte Person der Mittelschicht verfügt hier über einen Hochschulabschluss. Der Anteil der mittleren Bildung ist aber in Deutschland ebenso rückläufig wie der Anteil mit niedrigem Bildungsabschluss (vgl. Tabelle 3).

Analog zum Befund für die Mittelschicht verwundert es nicht, dass der Anteil der mittel- und hochqualifizierten Personen in der einkommensarmen Einkommensschicht

7 Als hohe Bildung gelten die ISECD 2011 Niveaus 5 bis 8, als mittlere Bildung die Niveaus 3 und 4 und als niedrige Bildung die Niveaus 0 bis 2 (Metis, 2022).

Tabelle 2
**Bevölkerungsanteile in verschiedenen Einkommensklassen**

| | Einkommensarm | Armutsgefährdet | Untere Mitte | Mittlere Mitte | Obere Mitte | Hohe Einkommen |
|---|---|---|---|---|---|---|
| Österreich | | | | | | |
| 2019 | 10,0 | 16,3 | 23,7 | 31,5 | 12,2 | 6,3 |
| 2015 | 8,7 | 19,4 | 21,9 | 32,2 | 11,7 | 6,1 |
| 2010 | 9,0 | 18,2 | 22,7 | 30,6 | 12,2 | 7,1 |
| 2005 | 7,6 | 18,1 | 24,3 | 32,5 | 11,7 | 5,8 |
| 2000 | 7,8 | 17,8 | 24,3 | 33,2 | 11,4 | 5,5 |
| Deutschland | | | | | | |
| 2018 | 10,0 | 18,2 | 21,9 | 31,6 | 11,0 | 7,4 |
| 2015 | 9,8 | 18,3 | 21,9 | 30,2 | 12,1 | 7,6 |
| 2010 | 9,4 | 19,6 | 21,0 | 31,0 | 11,6 | 7,4 |
| 2005 | 9,1 | 18,5 | 22,4 | 31,1 | 11,3 | 7,6 |
| 2000 | 7,4 | 17,2 | 25,5 | 32,9 | 10,9 | 6,2 |

Einkommensarm = weniger als 50 %, armutsgefährdet = 50 % bis 75 %, mittlere Einkommen = 75 % bis 200 %, hohe Einkommen = mehr als 200 %. Die Prozentzahlen beziehen sich jeweils auf den Median des äquivalisierten verfügbarem Haushaltseinkommen. Die angegebenen Werte bezeichnen jeweils den relativen Anteil in %.

Quelle: LIS Cross-National Data Centre (2022); eigene Berechnungen.

Tabelle 3
**Demografie, Haushaltsformen und formale Bildung von Personen in der Mittelschicht**

| | Österreich | | | Deutschland | | |
|---|---|---|---|---|---|---|
| | 2000 | 2010 | 2019 | 2000 | 2010 | 2018 |
| Demografie | | | | | | |
| 0 bis 20 Jährige | 22,2 | 20,9 | 19,5 | 21,5 | 18,3 | 18,3 |
| 20-30 Jährige | 13,3 | 12,4 | 11,5 | 9,8 | 9,3 | 8,9 |
| 30-40 Jährige | 17,4 | 12,3 | 14,3 | 18,1 | 11,6 | 11,0 |
| 40-50 Jährige | 15,0 | 19,5 | 13,7 | 15,8 | 18,6 | 13,3 |
| 50-60 Jährige | 13,2 | 14,1 | 16,9 | 12,9 | 15,8 | 18,1 |
| 60+ Jährige | 18,9 | 21,1 | 24,1 | 21,9 | 26,4 | 30,4 |
| Haushaltstypen | | | | | | |
| Einpersonenhaushalt | 11,2 | 12,3 | 13,7 | 13,8 | 15,5 | 16,4 |
| Paare ohne Kinder | 18,2 | 21,7 | 25,0 | 28,0 | 30,2 | 31,3 |
| Paare mit Kindern | 52,9 | 49,0 | 47,6 | 50,8 | 47,4 | 44,0 |
| Alleinerziehend mit Kindern | 6,9 | 5,1 | 3,8 | 3,9 | 4,5 | 4,7 |
| Sonstige | 10,8 | 11,9 | 9,8 | 3,5 | 2,5 | 3,5 |
| Formale Bildung | | | | | | |
| Niedrige Bildung | 24,2 | 22,3 | 15,2 | 14,4 | 12,5 | 11,7 |
| Mittlere Bildung | 67,0 | 61,7 | 67,0 | 62,5 | 60,1 | 56,8 |
| Hohe Bildung | 8,7 | 16,0 | 17,8 | 23,1 | 27,3 | 31,6 |

Die angegebenen Werte bezeichnen jeweils den relativen Anteil in %. Als hohe Bildung gelten die ISECD 2011 Niveaus 5 bis 8, als mittlere Bildung die Niveaus 3 und 4 und als niedrige Bildung die Niveaus 0 bis 2.

Quelle: LIS Cross-National Data Centre (2022), eigene Berechnungen.

in beiden Ländern steigt. So hatten in Österreich im Jahr 2000 nur 8,1 % eine formal hohe Bildung und 46,6 % einen mittleren Bildungsabschluss. Im Jahr 2019 waren schon 15,3 % hochqualifiziert und 53,4 % hatten einen mittleren Bildungsabschluss. In Deutschland ist die Tendenz nicht ganz so eindeutig. So ist unter den einkommensarmen Personen der Anteil von Hochqualifizierten von 11,2 % im Jahr 2000 auf 15,7 % im Jahr 2018 gestiegen, der Anteil mit mittlerem Bildungsabschluss ist aber leicht von 55,4 % auf 52,8 % zurückgegangen.

Gleichzeitig ist es für Personen mit niedriger oder mittlerer formaler Bildung immer schwieriger, sehr hohe Einkommen zu erzielen. In Österreich ist der Anteil von niedrig Qualifizierten zwischen 2000 und 2019 von 10,9 % auf 5,3 % zurückgegangen. Bei Personen mit mittlerer formaler Bildung sank der Anteil von 64,6 % auf 50,4 %. In Deutschland zeigt sich der gleiche Trend. Hatten im Jahr 2000 noch 6,4 % der Personen mit hohem Einkommen eine niedrige formale Bildung waren es 2018 nur noch 3,8 %. Bei Personen mit mittlerem Bildungsabschluss sank der Anteil von 44,8 % auf 33,1 %.

## Die Bedeutung der Mittelschicht im Sozialstaat

Nach der Vermessung der Mittelschicht in Deutschland und Österreich soll nun die Bedeutung der Mittelschicht für eine Gesellschaft dargelegt werden. Eine breite Mitte ist oftmals ein Indiz für einen stärkeren gesellschaftlichen Zusammenhalt eines Landes (Kelly, 2000; Lynch und Kaplan, 1997; Thorson, 2014). Auch die Einkommensmobilität eines Landes profitiert von der Breite der Mittelschicht (OECD, 2018). Einen wesentlichen Einfluss auf die Durchlässigkeit hat dabei der Sozialstaat. Zum einen ist die Progression bei den Steuern und Abgaben dafür ausschlaggebend, inwiefern sich höhere Löhne auch in gestiegenen verfügbaren Haushaltseinkommen niederschlagen. Zum anderen unterstützt der Staat mit Geld und Sachleistungen und erhöht damit bei den Rezipient:innen der Transfers das verfügbare Einkommen.

Betrachtet man in beiden Ländern nur die monetären Ströme, so zeigt sich ein eindeutiger Befund. Unter der ausschließlichen Berücksichtigung von direkten Steuern und Abgaben sowie den monetären Transferzahlungen zählt die Mittelschicht in Deutschland als Nettozahlerin (OECD, 2021a). Die detaillierte Analyse für Österreich bestätigt diesen Befund auch für die Alpenrepublik. Auch hier übersteigen die direkten Steuern und Abgaben die monetären

Abbildung 3
**Steuern und Abgaben sowie monetäre Transferleistungen in Österreich**
in Euro

Sonstige Geldtransferleistungen umfassen Familienleistungen, Wohnbeihilfe und Geldleistungen für Gesundheit. Direkte Steuern beinhalten Steuern auf Arbeit, Kapitalerträge und Vermögenseinkommen.

Quelle: Christl et al. (2020).

Abbildung 4
**Direkte und indirekte Steuern und Abgaben sowie monetäre Transferleistungen in Österreich**
in Euro

Sonstige Geldtransferleistungen umfassen Familienleistungen, Wohnbeihilfe und Geldleistungen für Gesundheit. Direkte Steuern beinhalten Steuern auf Arbeit, Kapitalerträge und Vermögenseinkommen. Zu den indirekten Steuern zählen Steuern auf Konsum (z. B. Mehrwert- und Tabaksteuer).

Quelle: Christl et al. (2020).

Transferleistungen der Mittelschicht (Perzentile 26 bis 93) deutlich (vgl. Abbildung 3) (Christl et al., 2022b).

Etwas differenzierter wird das Bild, wenn auch indirekte Steuern sowie Sachleistungen wie Bildung und Gesundheit Berücksichtigung finden (vgl. Abbildung 4). Insgesamt stellt sich auch hier die Mittelschicht als Nettozahlerin heraus, allerdings zählt die untere Mittelschicht noch zu den Nettoempfängern.

Eine entscheidende Rolle spielt der Sozialstaat auch bei den Auswirkungen der Coronapandemie für die Mittelschicht. Die Coronakrise führte in (fast) allen Ländern der Welt zu Wohlstandsverlusten (OECD, 2021b). In Deutschland und Österreich konnte der Sozialstaat allerdings die Auswirkungen des Wirtschaftseinbruchs auf die Haushaltseinkommen zu einem erheblichen Teil mildern. Dies liegt zu großen Teilen an den automatischen Stabilisatoren, die Einkommensverluste infolge des Jobverlusts deutlich abfedern.[8]

Der Coronaschock wirkte in beiden Ländern regressiv auf die Einkommensverteilung. Christl et. al (2022a) zeigen für Österreich und Christl et al. (2022b) für Deutschland, dass durch die ergriffenen Maßnahmen aber die Einkommenseinbußen weitestgehend ausgeglichen werden konnten. Aufgrund der Einmalzahlung für Arbeitslose konnten sich Personen im untersten Einkommensdezil in Österreich sogar verbessern.

Im ersten Jahr der Pandemie 2020 kam insbesondere auch der Kurzarbeit eine tragende Rolle zur Erhaltung der Kaufkraft der Beschäftigten zu. In diesem Zeitraum stieg in Deutschland die Zahl der Menschen in Kurzarbeit auf über 6 Mio. an, in Österreich waren es mehr als 1 Mio. Arbeitnehmer:innen (Christl et al., 2022a; Christl et al., 2022b). Zusätzlich wurde in beiden Ländern eine Einmalzahlung für Kinder getätigt. Weitere Sozialleistungen waren eine Steuererleichterung für Alleinerziehende in Deutschland oder Einmalzahlungen an Arbeitslose in Österreich.

## Herausforderungen für eine stabile Mittelschicht

Wenngleich sich die Mittelschicht in Österreich und Deutschland in den vergangenen Jahrzehnten als überaus widerstandsfähig erwiesen hat, so gibt es dennoch einige Entwicklungen und Trends abseits der Coronapandemie und des aktuellen Kriegsgeschehens, die eine Herausforderung für die Resilienz der Mittelschicht darstellen. Zukünftig wird es für Menschen ohne entsprechende Qualifikation vermutlich immer schwieriger, mittlere oder hohe Einkommen zu erzielen. Zwar ist eine gute formale Ausbildung keine Garantie für ein höheres Einkommen mehr, aber ohne Bildung ist dies fast ausgeschlossen. Dies gilt insbesondere vor dem Hintergrund

8 Die Wirkungsmächtigkeit der automatischen Stabilisatoren liegt in Deutschland knapp über EU-Durchschnitt und in Österreich mit Rang sieben von 27 EU-Mitgliedstaaten deutlich darüber (Mourre et al., 2019).

eines immer vernetzteren und digitalisierteren Wirtschaftslebens.

Auch die demografische Entwicklung wird zu einer Herausforderung. Die Gesellschaften in Deutschland und Österreich werden unaufhaltsam älter. Während in Österreich die demografische Situation durch Zuwanderung ein wenig entschärft werden konnte, ist dies in Deutschland nicht zuletzt wegen der Größe nicht der Fall. Diese Verschiebung wird zwar einerseits zu einer besseren Arbeitsmarktsituation der jungen Generation führen, andererseits werden aber gleichzeitig die Ausgaben für Renten, Gesundheit und Pflege ansteigen. Hinzu kommt, dass alternde Volkswirtschaften meist weniger dynamisch sind und mit geringerer gesamtwirtschaftlicher Produktivität und einem geringeren Wachstum einhergehen (Bloom et al., 2000; Persson, 2004; oder Aiyar und Ebke, 2016).

Damit die Mittelschicht in Deutschland und Österreich auch zukünftig stark und resilient bleibt, darf die Wirtschaftspolitik diese Herausforderungen nicht aus den Augen verlieren. Lebenslange (Weiter-)Bildung muss möglich sein und auch gefördert werden. Ebenso sollten Anstrengungen ergriffen werden, damit das Arbeitsangebot breiter wird. Wenn eine immer kleiner werdende Mittelschicht die großen demografischen Lasten schultern muss, besteht die Gefahr, dass diese auf Dauer immer mehr erodiert.

**Title:** ***The Development of the Middle Class in Austria and Germany***
